# PFAS Contamination and the Impacts of Environmental Turbulence: The Role of Collective Memory and Narrative Epidemiology in Invisible Disaster

**DOI:** 10.3390/ijerph23040448

**Published:** 2026-03-31

**Authors:** Marialuisa Menegatto, Michael R. Edelstein, Danielle DeVasto, Adriano Zamperini

**Affiliations:** 1 Department of Philosophy, Sociology, Pedagogy and Applied Psychology, University of Padova, 35131 Padova, Italy; 2 Interuniversity Research Centre in Environmental Psychology (CIRPA), 00185 Rome, Italy; 3 School of Social Science and Human Services, Ramapo College of New Jersey, Mahwah, NJ 07430, USA; medelste@ramapo.edu; 4 Department of Writing, Grand Valley State University, Allendale, MI 49401, USA; devastod@gvsu.edu

**Keywords:** chronic environmental contamination, psychosocial impact, narrative epidemiology, collective memory, technological disaster, public health

## Abstract

**Highlights:**

**Public health relevance—How does this work relate to a public health issue?**

- PFAS contamination is framed as a slow invisible chronic technological disasters producing long-term psychosocial health effects beyond toxicological exposure alone.
- The study shows that environmental contamination reshapes risk perception, trust in institutions, and collective behavior, all of which are key determinants of population health outcomes.

**Public health significance—Why is this work of significance to public health?**

- By integrating Environmental Turbulence Theory with narrative epidemiology, the study expands public health assessment beyond biomedical indicators to include psychosocial dimensions of harm.
- The findings demonstrate that psychosocial impacts, such as chronic stress, institutional distrust and stigma, are structural components of environmental health crises and influence community resilience and recovery.

**Public health implications—What are the key implications or messages for practitioners, policy makers and/or researchers in public health?**

- Public health responses to chronic contamination should adopt transdisciplinary approaches combining epidemiological data with lived experience and narrative-based evidence.
- Psycho-Social Impact Assessment (PSIA) can be used as an operational tool to identify hidden suffering and support more responsive environmental health policies and community interventions.

**Abstract:**

PFAS contamination represents a slow, invisible chronic technological disaster with documented long-term psychosocial impacts on affected communities. However, existing research has predominantly focused on toxicological and biomedical outcomes, leaving the lived experiences and narrative dimensions of contamination underexplored. This study investigates how residents of PFAS-contaminated communities experience and narrate environmental contamination by applying Edelstein’s Theory of Environmental Turbulence (TET) and integrating a bottom-up stage-based model of psychosocial reaction with narrative epidemiology. Twenty-five personal narratives were selected from the digital archive Living With PFAS and analyzed through thematic analysis. Three main themes emerged, corresponding to the TET dimensions of lifescape, lifestyle, and lifestrain, articulated across twelve subthemes: inversion of health, self, home community and place, environment, livelihood, trust, environmental stigma, shock and fear, chronic concern, anger, parental guilt and relation strain. The findings demonstrate that PFAS contamination produces multidimensional disruptions that extend beyond toxic exposure to encompass identity, social relationships, institutional trust, and collective memory. Integrating TET with Psycho-Social Impact Assessment (PSIA) offers a theoretically grounded and exploratory transdisciplinary framework for identifying hidden suffering and informing more responsive environmental health policies and community interventions.

## 1. Introduction

One of the earliest toxic disasters to receive global notoriety was the 1976 explosion at a pharmaceutical plant in Seveso, northern Italy, which released a toxic cloud of dioxin over the surrounding area. The accident represents a classic example of an acute technological disaster that later evolved into a chronic environmental crisis affecting the surrounding community [1,2]. Although the toxic substance involved was invisible, the event itself had high public visibility and rapidly became a symbol of industrial risk.

In contrast to such acute events, many contemporary environmental crises unfold gradually and often remain undetected for long periods. Increasing attention has therefore shifted toward a new generation of persistent synthetic chemicals that have significantly expanded human chemical exposure [3,4]. Among these substances, per- and polyfluoroalkyl substances (PFAS) represent a particularly concerning class of contaminants due to their widespread use, environmental persistence, and potential health impacts [5,6,7,8].

PFAS are a large class of persistent synthetic chemicals widely used in industrial and consumer products. Their extraordinary chemical stability has led to their persistence in the environment and their accumulation in human and animal tissues [9], earning them the designation “forever chemicals” [10]. As a result, PFAS contamination has emerged as a major environmental and public health issue affecting communities worldwide with pervasive environmental distribution and documented health impacts across populations [11]. Exposure to certain PFAS compounds has been associated with a range of adverse health outcomes [11,12,13]. Epidemiological and clinical studies have reported links between PFAS exposure and increased cholesterol levels [14], kidney cancer and thyroid disease [15], cardiovascular toxicity and the promotion of atherosclerosis as well as reduced immune response [16], and elevated risks of testicular cancers [17]. More recent research has also highlighted potential associations with metabolic disorders and endocrine-disrupting effects [18], reproductive outcomes [19,20], and developmental outcomes in exposed populations, further reinforcing concerns about the long-term health impacts of PFAS exposure [13]. Living in PFAS-contaminated areas has been linked to chronic psychological distress and persistent concern about long-term health effects [21,22,23,24,25].

The focus of this study is on communities experiencing localized environmental contamination. In such contexts, exposure often results from long-term industrial emissions that contaminate water sources, soil, and food chains. Unlike acute industrial accidents, PFAS contamination typically develops as a slow, invisible, and long-lasting environmental process that may remain undetected for years or even decades.

These characteristics place PFAS contamination within the category of what can be described as a “slow invisible chronic technological disaster”. In such situations, the absence of a clearly identifiable event, the delayed recognition of exposure, and the uncertainty surrounding health consequences create unique social and psychological challenges for affected communities [26,27,28].

Previous environmental disasters have shown that long-term contamination can profoundly disrupt community life and undermine trust in institutions. The Love Canal disaster in the United States, for example, became a defining case of the “contaminated community”, demonstrating how long-term exposure to industrial pollutants can produce not only environmental and health problems but also social conflict, uncertainty, and loss of trust in institutions [29,30]. Similar dynamics have emerged in other cases of PFAS contamination, such as the well-known PFOA contamination linked to industrial emissions in Parkersburg, West Virginia [31,32], in Pease Tradeport in Portsmouth, New Hampshire, and Hyannis Massachusetts [33], in Veneto Region, Italy [23,34,35].

Although the scientific literature on PFAS has grown rapidly, most studies have focused on toxicology [13], environmental monitoring [36,37], and epidemiological health outcomes [38], while comparatively less attention has been paid to the lived experiences of affected communities and how they interpret and narrate environmental contamination. Yet these social and narrative dimensions are crucial for understanding the broader public health implications of environmental crises.

To address this gap, this study draws on the Theory of Environmental Turbulence (TET) [39] which conceptualizes environmental contamination as a disruption of the fundamental relationships between communities and their ecological surroundings.

The analysis also incorporates a stage-based model of psychosocial reaction to toxic exposure developed through a bottom-up approach [23] which derives its conceptual categories from the lived experiences of affected populations. In this sense, the model is consistent with the perspective of narrative epidemiology, which emphasizes the role of personal and collective narratives in revealing patterns of exposure, illness, and social meaning within affected communities.

This study investigates how residents of PFAS-contaminated communities experience and narrate environmental contamination. Drawing on Environmental Turbulence Theory with narrative epidemiology, it examines how personal testimonies contribute to the construction of collective memory and to understanding the psychosocial impacts of slow invisible chronic technological disasters. In this perspective, narratives are not only complementary to epidemiological data, but constitute an independent epistemic resource through which patterns of exposure, interpretation, and response become visible. In addition, this study draws on a bottom-up stage-based model of psychosocial reaction, which allows us to examine how such disruptions unfold over time, thereby integrating structural and temporal dimensions of environmental turbulence.

## 2. Collective Memory of Technological Disaster and Narrative Epidemiology

The collective memory of environmental and technological disasters is often shaped by their visibility, immediacy, and emotional impact [40,41,42]. Acute catastrophes such as the Bhopal tragedy, the Seveso incident, and the Chernobyl nuclear disaster are deeply embedded in global memory because of their sudden onset, dramatic imagery, and intense media coverage. These events produced powerful symbols, such as the abandoned city of Pripyat near Chernobyl [43], the mass casualties in Bhopal [44], or the evacuated towns and children affected by chloracne in Seveso [1,45,46,47], that became central to national and international consciousness.

In contrast, slow invisible chronic technological disasters rarely enter collective memory in the same way. They typically develop gradually, remain undetected for long periods. As a result, public recognition may be delayed and affected communities may experience prolonged uncertainty while institutions investigate or contest the extent of contamination [26,28]. Without dramatic imagery or a clearly defined event, such disasters often remain marginal in broader public memory despite their long-term environmental and health consequences [48].

Nevertheless, some contamination events have achieved broader visibility through community mobilization, media attention, and litigation. The Love Canal disaster in the United States became a defining example of a “contaminated community”, eventually leading to the creation of the federal Superfund program [49,50]. Similarly, PFAS contamination associated with industrial emissions in the Veneto region in Italy—estimated to have begun in the 1960s but only recognized later—has progressively attracted public and institutional attention since 2013 through scientific investigation, media reporting, and legal proceedings [35,51,52,53]. Despite this attention, PFAS contamination often remains marginal in broader collective memory, even though it affects numerous communities and persists in the environment for decades. For residents of contaminated areas, the delayed recognition of exposure often produces what has been described as the “shock of discovery”, the realization that one’s environment and body have been silently contaminated for years [23,54]. Such experience can generate feelings of betrayal, fear, and loss of control particularly when exposure results from industrial activities or regulatory failures. In some cases, affected communities may also encounter skepticism or denial from institutions and the broader public, a process described as “second-order victimization”, in which the suffering of victims is questioned or minimized [55,56].

These dynamics cannot be fully understood without considering the narrative dimension of collective memory. Following Halbwachs’ foundational work [40], memory is not a passive record of past events but a social process through which communities interpret and transmit experiences across generations. Narratives play a central role in this process by organizing events into meaningful stories that connected causes, consequences, and moral interpretations. This narrative function becomes particularly important in slow invisible chronic technological disasters, where harm accumulates gradually and lacks a clearly identifiable beginning. By transforming fragmented experiences into coherent accounts, storytelling enables individuals and communities make sense of complex events and integrate them into collective memory [57,58]. Through the sharing of testimonies, narratives can also foster solidarity and contribute to the formation of cultural memory transmitted through public discourse, media coverage, and institutional debates [59,60].

In recent years, the concept of narrative epidemiology has highlighted the importance of personal testimonies and lived experiences in understanding patterns of exposure, illness, and risk perception within affected populations. While traditional epidemiology, much as behaviorist-based social science, focuses on quantifying disease patterns and identifying causal relationships, it often lacks the tools to capture the psychosocial, experiential, emotional, and symbolic dimensions of illness, especially in communities affected by long-term, imperceptible toxic exposure [61,62,63,64]. Narrative approaches complement conventional epidemiological methods by illuminating these social and emotional dimensions of environmental crises that are often overlooked in purely biomedical analyses.

For slow invisible chronic technological disaster, narrative epidemiology becomes especially important. It offers a crucial bridge between empirical data and lived human experience. It addresses the profound challenges posed by environmental threats that lack immediate sensory markers, such as those caused by PFAS contamination. It helps uncover the hidden trajectories of exposure and illness that traditional statistical and epidemiological methods may overlook. As survivors articulate their experiences, they contribute to the formation of a ‘narrative diagnosis’ [61], a communal process through which fragmented memories are woven into coherent accounts that can influence public awareness, shape policy responses, and demand justice. This process is not only therapeutic but also political, as it contests official narratives of the event and asserts alternative forms of knowledge grounded in lived experience. Such narratives that detail the lived experience of environmental contamination are not merely anecdotal supplements to scientific data, but constitutive elements in the ways risks are perceived, memories are formed, and collective identities are redefined [65].

These perspectives align closely with Environmental Turbulence Theory (TET), which conceptualizes environmental contamination as a disruption of the fundamental relationships between individuals, communities, and their ecological surroundings. According to TET, technological disasters can produce profound psychosocial consequences by destabilizing daily routines, social relationships, and systems of meaning [66]. Within this framework, narratives of contamination provide valuable insights into how affected communities interpret environmental disruption, make sense of their experiences and reconstruct collective memory in the aftermath of slow and invisible disasters.

## 3. The Theory of Environmental Turbulence (TET)

This study aligns with the research on the psychosocial impacts experienced by communities exposed to environmental or technological disaster. These studies highlight how such events affect not only material conditions or physical health but also the social and emotional fabric of everyday life [23,66,67,68]. Environmental contamination influences how individuals perceive their health, their relationships with others, their surroundings, and their expectations for the future.

To better understand these complex psychosocial disruptions, the Padua research group contextualized its analysis within Michael R. Edelstein’s Theory of Environmental Turbulence (TET) [39,66]. TET conceptualizes environmental contamination as a disruption of the normal relationships between individuals, communities, and their ecological surroundings. Originally developed through narrative analyses of communities affected by environmental harm, the theory provides a framework for understanding how people interpret and respond to both acute and prolonged environmental stressors.

According to Edelstein, environmental turbulence reverberates across a social process continuum linking individual experiences with broader community and societal dynamics. Individual responses are embedded within collective processes and interact with social institutions and cultural interpretations of environmental change. To analyze these impacts, TET identifies three interrelated dimensions, lifescape, lifestyle, and lifestrain which respectively refer to disruptions in fundamental meanings, changes in everyday practices, and the psychosocial strain associated with environmental contamination.

Lifescape refers to disruptions in fundamental understandings of health, identity, place, and trust. In contaminated environments, previously taken-for-granted assumptions about safety and normalcy are destabilized. Home may shift from a place of security to a potential source of exposure, while the surrounding environment becomes perceived as threatening rather than protective. At the same time, trust in institutions, scientific expertise, and regulatory authorities may be undermined, contributing to broader processes of environmental stigma and social uncertainty.

Lifestyle refers to changes in daily routines and everyday practices resulting from environmental contamination. Residents may alter their behaviors to reduce exposure, such as relying on bottled water, limiting contact with contaminated soil, or modifying domestic routines. These adjustments can disrupt established patterns of everyday life and serve as continuous reminders of the underlying environmental threat. In contaminated communities, residents may also devote substantial time and energy to meetings, advocacy, and collective actions aimed at addressing the crisis.

Lifestrain captures the psychological and social stress associated with environmental disruption. Emotional responses may include fear, grief, anger, and a sense of injustice, while uncertainty about long-term health effects can generate persistent anxiety and loss of control. At the social level, these stresses may strain relationships, erode trust in institutions, and contribute to feelings of marginalization or stigma. Lifestrain therefore reflects the broader impact of environmental turbulence on mental health, well-being, and social cohesion.

## 4. Materials and Methods

### 4.1. Aim and Rationale

Using the interpretative lens of the Theory of Environmental Turbulence, research was undertaken to identify core themes related to the collective memory and narrative epistemology of slow invisible chronic technological disasters resulting from PFAS contamination. Edelstein operationalized these theoretical insights through Psycho-Social Impact Assessment (PSIA), a qualitative methodological approach designed to document and interpret the psychosocial consequences of environmental disruption [65]. PSIA relies on in-depth interviews with affected residents and preserves their accounts as narrative testimonies that can be analyzed for recurring themes and patterns. These narratives are then triangulated with direct observation, documentary evidence, and other sources to reconstruct the transition from pre-contamination conditions to post-contamination experiences. In this way, PSIA provides practical analytical tools for examining how environmental turbulence manifests in lived experience.

This theoretical framework provides the analytical lens through which the narratives collected in this study are interpreted, allowing for the identification of disruptions across lifescape, lifestyle, and lifestrain in contexts of environmental contamination.

With specific reference to our case study, we posed the following research questions: What forms, modes, and dimensions does the collective remembering of a slow and invisible disaster take? What epidemiological narratives can bridge empirical data and lived human experience? While our analysis focused specifically on PFAS contamination, the findings may offer valuable insights for future research in the broader field of collective memory, epidemiology, and public health as they pertain to slow, invisible chronic technological disasters. Additionally, the results provide practical recommendations for more effective collective responses to environmental toxic contamination challenges.

### 4.2. Methodological Approach

This study adopts a qualitative methodological approach based on the analysis of narrative testimonies collected in a public archive in order to capture the lived experiences of residents exposed to long-term environmental contamination. Qualitative approaches are particularly appropriate for investigating the psychosocial impacts of environmental crises because they allow researchers to explore perceptions, meanings, and everyday practices that are often difficult to capture through quantitative indicators alone [69]. In recent years, qualitative and narrative methods have been increasingly used in environmental health research to examine how communities interpret environmental risk, contamination, and institutional responses [70]. For example, recent qualitative studies have explored the lived experiences and psychosocial consequences of PFAS contamination in affected communities using interviews and narrative analysis, demonstrating the relevance of these approaches for understanding how residents experience chronic exposure and adapt to environmental disruption [23]. Systematic reviews of research on PFAS-exposed communities have similarly highlighted the importance of documenting lived experiences in order to capture the social, emotional, and institutional dimensions of environmental contamination that may remain invisible in purely biomedical or toxicological analyses [54]. More broadly, recent environmental health research continues to emphasize the value of qualitative and mixed-method approaches for investigating community perceptions of environmental hazards and their implications for health and wellbeing [71]. Within this framework, narrative testimonies are treated not only as qualitative data but also as epistemic resources that reveal how individuals and communities interpret environmental disruption. The analysis followed a bottom-up, inductive approach [23], in which themes were generated directly from the narratives through an iterative coding process, rather than being predefined according to the theoretical framework. This allowed the identification of recurring patterns grounded in participants’ lived experiences. The Theory of Environmental Turbulence (TET) was subsequently applied as an interpretive lens to organize and theoretically contextualize the emergent themes, enabling a deeper understanding of how disruptions unfold across lifescape, lifestyle, and lifestrain.

In line with the perspective of narrative epidemiology, these accounts provide insight into how experiences of contamination are socially constructed and collectively interpreted, thereby offering an empirical basis for examining, and empirically grounding, the psychosocial dynamics described by Environmental Turbulence Theory.

### 4.3. Research Design

An archive of narrative data relating to PFAS contamination was selected as the subject of analysis. The archive was collected through ‘Living with PFAS’ project [72], directed by Dr. Danielle DeVasto (Grand Valley State University) and contained in a digital public archive [73] dedicated to documenting and preserving the stories of individuals, communities, and ecosystems affected by PFAS. The project’s mission is to create a platform for sharing collective experiences that can inform future action and decision-making recognizing that technical data alone are insufficient to capture the full impact of PFAS. The archive was created as part of a community-based initiative aimed at documenting the experiences of residents affected by PFAS contamination.

The archive was selected for several reasons. First, by gathering and preserving personal accounts, Living with PFAS functions not only as a research tool but also as a site of collective memory. It contributes to the construction of a shared historical record that gives voice to communities often marginalized in official narratives. In this sense, the archive becomes a means of acknowledging lived experiences, fostering social recognition, and sustaining public memory around environmental injustice and its long-term consequences. Second, the archive was chosen for its rich and specific narrative content. The narratives offer first-hand perspectives from those most affected by PFAS, addressing themes such as risk perception, trust in institutions, health-related anxiety, and broader social repercussions. These dimensions are crucial for understanding the psychosocial impact of environmental contamination and provide a necessary complement to the more technical and scientific analyses typically associated with PFAS studies.

The testimonies analyzed in this study were publicly shared by contributors within the archive. For research purposes, the narratives were examined in anonymized form and no identifying information was reported.

### 4.4. Materials and Participants

The Padua group selected 25 narratives of individuals who had been exposed directly to PFAS contamination, 9 males and 16 females, from the ‘Living with PFAS’ online archive. Because the testimonies were produced through spontaneous participation rather than through a structured recruitment process, the study does not employ a conventional sampling strategy [74,75]. Instead, the authors selected 25 narratives from the archive based on qualitative relevance criteria. Narratives were included in the analysis if they (1) described personal or family experiences related to PFAS contamination, (2) provided sufficiently detailed accounts of everyday life, perceptions of environmental risk, or interactions with institutions, and (3) were complete and accessible within the archive. The aim of the selection was therefore not statistical representativeness but the identification of narratives rich in experiential content suitable for qualitative and narrative analysis.

Their ages ranged from 35 to 69 (*M* = 55.47) (*n* = 5 missed cases for which age information was not available). Demographic information associated with the narratives (such as age) was available only when voluntarily provided by contributors at the time of submission to the archive. As a result, some testimonies did not include complete demographic information. In five cases, the age of the contributor was not specified in the original record. However, since the analysis focuses on the narrative content of the testimonies rather than on demographic comparisons, these omissions do not affect the qualitative interpretation of the data. Duration of participants’ exposure to PFAS contamination ranged from 7 to 69 years (see Table 1). The narratives were selected, numbered and transcribed verbatim and between September 2023 and September 2024.

### 4.5. Data Analysis

The Padua team conducted a thematic analysis of the narratives obtained from the archive, following a multi-phase, iterative process [74,76]. The initial stage involved an in-depth familiarization with the narrative transcripts through careful and repeated reading. Two researchers independently applied open coding, generating preliminary codes and identifying emerging thematic elements. The coding results were then compared and discussed collaboratively in order to identify convergences and discrepancies in interpretation. When differences emerged, they were addressed through iterative discussion until a shared interpretation was reached. To improve consistency and reliability, regular meetings were held to review and refine the coding framework. This iterative dialogue allowed the coding categories to be progressively clarified and stabilized as the analysis advanced. The analysis followed an abductive and iterative logic [77,78], characterized by a constant movement between participants’ narratives and emergent analytical categories. This approach allowed us to remain grounded in the data while also engaging critically with theoretical concepts. By balancing inductive reasoning, from data to theory with deductive processes, from theory to data, both the analytical rigor and the generative potential of the research were ensured [79]. This collaborative and reflexive analytical process contributed to strengthening the credibility and trustworthiness of the qualitative interpretation. The qualitative software package Atlas.ti (version 9.1.7, Scientific Software Development GmbH, Berlin, Germany) was used to facilitate data analysis [35]. Because the narratives analyzed in this study were drawn from a pre-existing archive of voluntary testimonies, data collection did not follow an iterative process aimed at reaching theoretical saturation, as is common in interview-based qualitative research [74]. Instead, the selection focused on narratives that provided rich descriptions of lived experiences related to PFAS contamination. During the analytical process, recurring thematic patterns emerged across the narratives, suggesting a level of thematic convergence that supported the interpretative robustness of the analysis.

## 5. Results

As shown in Table 2, the results of this study are grouped into the following three themes, which describe the main dimensions of the Theory of Environmental Turbulence as applied to a PFAS contamination along with twelve subthemes. The results are presented through a thematic and interpretative analysis of the narratives, in which individual testimonies are used as illustrative examples of broader patterns related to environmental turbulence.

### 5.1. Theme 1: Lifescape

This theme captures the disruption of the lifescape, understood as the underlying framework through which individuals interpret safety, stability, and everyday life. Across a substantial portion of the narratives (*n* = 10), participants describe a profound reconfiguration of taken-for-granted assumptions about their environment, particularly following the discovery of PFAS contamination. In line with Environmental Turbulence Theory, this moment of realization represents a critical rupture in which previously stable meanings become visible precisely because they are destabilized.

In many accounts, this disruption emerges through what has been described as the “shock of discovery”, when individuals first become aware of contamination and begin to reassess their past experiences in light of new information. This process often involves a retrospective reinterpretation of everyday practices—such as drinking water—that were previously perceived as safe and routine. As one participant explains:

*“I like to say in May of 2014, my life changed forever. I read a newspaper article that said they found high levels of PFAS in the drinking water wells at the Pease Tradeport that is home to a former Air Force base that had been shut down in 1991.”*

In this case, the discovery of contamination triggers a fundamental shift in meaning: water, previously taken for granted as a neutral and life-sustaining resource, is redefined as a source of invisible risk. Similar reinterpretations appear across multiple narratives in the dataset, where ordinary elements of daily life, home, water, and environment, are transformed into potential threats. This inversion reflects a core lifescape dynamic: the erosion of basic assumptions of safety and predictability. The environment is no longer experienced as a stable backdrop but as an uncertain and potentially harmful surround, requiring constant reinterpretation. In this sense, PFAS contamination does not only introduce a physical hazard but fundamentally alters the cognitive and symbolic structures through which individuals make sense of their everyday lives.

#### 5.1.1. Inversion of Health

This subtheme illustrates a key lifescape transformation: the inversion of health from a taken-for-granted condition into a source of ongoing uncertainty and concern. Across several narratives (*n* = 9), participants describe a shift in which health is no longer perceived as stable but as fragile, unpredictable, and potentially compromised by long-term exposure to PFAS.

This transformation is closely linked to the reinterpretation of both personal and collective experiences of illness. Participants frequently report a heightened awareness of disease within their communities, often describing what they perceive as an unusual concentration of severe health conditions. As one participant reflects: “I looked around and thought… there’s just too much illness, too much pain”.

In these accounts, illness is no longer understood as an isolated or random event but becomes part of a broader narrative of environmental contamination. This shift reflects a fundamental reconfiguration of causal reasoning, in which previously unrelated health events are retrospectively connected to exposure. At the individual level, this inversion is further reinforced by personal experiences of illness that are interpreted through the lens of contamination. One participant describes the emergence of multiple severe health conditions following exposure:

*“I developed ovarian cysts the size of oranges… and my hormone levels are completely out of balance. And I have high cholesterol. I never had any of this, years ago.”*

Such narratives illustrate how bodily changes are reinterpreted as evidence of environmental harm, contributing to a loss of confidence in one’s own physical integrity. Within the framework of TET, this reflects a disruption of lifescape at the level of core assumptions about health, the body, and vulnerability.

In addition to retrospective reinterpretation, participants also express persistent uncertainty regarding future health outcomes. Concerns about potential long-term effects, often extending to newer forms of contamination such as short-chain PFAS, generate a continuous sense of anticipatory anxiety. As one participant states:

*“Will we wake up in five or ten years and have major medical issues… I don’t want to deal with another trauma.”*

This projection of risk into the future further destabilizes the lifescape by extending uncertainty beyond the present, creating a condition in which health is experienced not as a stable state but as an ongoing and unresolved threat. In this sense, PFAS contamination reshapes not only perceptions of current well-being but also expectations about the future, embedding vulnerability into everyday life. This inversion of health also reflects a broader epistemic shift, in which uncertainty replaces biomedical certainty and lay interpretations of exposure gain centrality in making sense of illness.

#### 5.1.2. Inversion of Self

This subtheme captures a further transformation of the lifescape: the erosion of personal control and agency in the face of environmental contamination. Across multiple narratives (*n* = 11), participants describe a shift from acting autonomously to reacting under conditions of uncertainty, often becoming dependent on external expertise, institutional decisions, and incomplete or contested information. This loss of control frequently emerges at the moment of discovery, when individuals are confronted with a threat they do not understand and feel unable to manage. As one participant recalls: “What is this? What does it do? What does this mean?”

Such expressions reflect not only a lack of information but a deeper disruption of the self, in which the capacity to interpret and respond to one’s environment is suddenly undermined. Within the framework of TET, this represents a core lifescape inversion: the familiar world becomes uncertain and cognitively unmanageable, and individuals experience themselves as lacking the resources to act effectively.

This sense of powerlessness is further reinforced by the perceived irreversibility and scale of contamination. Participants frequently describe environmental damage as uncontrollable and beyond remediation, contributing to a sense of resignation and diminished agency. As one interviewee states: “Once the contamination is in the aquifer… there’s no putting it back in”.

In these narratives, the impossibility of restoring prior conditions translates into a loss of future-oriented control, where individuals feel unable to influence either environmental outcomes or their own long-term well-being. At the same time, uncertainty surrounding health consequences and the lack of clear institutional responses intensify this dynamic. Participants report feeling “out of answers” and unable to identify effective courses of action, highlighting a condition in which decision-making is constrained by ambiguity and dependence on external authorities.

This erosion of agency is not only individual but also collective. Several narratives describe a broader social climate characterized by confusion, fear, and conflicting information: “People are afraid. People are stressed. People don’t know what to believe”.

Such conditions further destabilize the lifescape by undermining shared frameworks of interpretation and trust. In this sense, PFAS contamination produces not only environmental and health uncertainty but also a crisis of agency, in which individuals and communities struggle to regain control over their present and future.

#### 5.1.3. Inversion of Home, Community and Place

This subtheme highlights a central lifescape inversion: the transformation of the home from a place of safety into a site of potential exposure. Across several narratives (*n* = 8), participants describe how PFAS contamination undermines the symbolic and material boundaries that traditionally define domestic security. What was once perceived as a protected and stable environment is reinterpreted as permeable, uncertain, and potentially harmful. This transformation is often triggered by the discovery of contamination in domestic water sources. In response, participants report implementing mitigation strategies, such as installing filtration systems, in an attempt to regain control over their immediate environment: “We installed a full house filtration system… a reverse osmosis system in the kitchen”.

However, these adaptive measures do not fully restore the sense of safety associated with the home. Instead, they reinforce the awareness that protection now depends on continuous intervention, rather than being an inherent property of the domestic space.

A key element emerging across narratives is the collapse of boundaries between inside and outside. Contamination is frequently described as a diffuse and mobile threat that crosses physical limits and infiltrates both private and surrounding spaces. As one participant recounts: “PFAS foam… came right up over our seawall and plopped in our backyard”. Similarly, others describe contamination maps showing pollutants extending through residential areas, including their own properties, reinforcing the perception that exposure cannot be spatially contained.

Within the framework of TET, this reflects a profound disruption of lifescape: the home loses its function as a secure anchor of everyday life and becomes part of a broader contaminated landscape. This shift not only alters the material conditions of living but also destabilizes the symbolic meaning of home as a space of protection, privacy, and control.

In this sense, PFAS contamination produces a form of “domestic uncertainty,” in which the boundaries between safe and unsafe, inside and outside, are blurred. The result is a persistent condition of vulnerability that extends beyond the individual dwelling to encompass the wider environment in which everyday life unfolds.

#### 5.1.4. Inversion of Environment

This subtheme reflects a further lifescape transformation: the inversion of the environment from a stable and benign backdrop into a source of diffuse and unpredictable threat. Across several narratives (*n* = 10), participants describe how PFAS contamination alters their relationship with the natural world, undermining long-standing assumptions about environmental safety, continuity, and control. This shift is particularly evident in accounts that emphasize the deep cultural and generational ties between individuals and their local environment. For some participants, contamination threatens not only ecological integrity but also identity, as place-based practices, such as farming, fishing, or land stewardship, are redefined as potentially hazardous. As one participant explains: “We don’t know where it’s going to end up… All we know is it’s going to keep spreading”.

In these narratives, the environment is no longer experienced as a predictable system but as a dynamic and uncertain process, characterized by invisible pathways of contamination that exceed individual and community control. This uncertainty disrupts the capacity to plan for the future and to maintain continuity with past practices, weakening forms of environmental knowledge transmitted across generations.

A recurring element across accounts is the perception of contamination as pervasive and boundaryless. Exposure is no longer associated with specific locations or behaviors but is instead understood as embedded in everyday life: extending to water, food, consumer products, and the broader ecosystem. As one participant states: “It’s in your food, it’s in your clothing… nobody is exempt”. This expansion of perceived risk contributes to a generalized sense of environmental insecurity, in which the distinction between safe and unsafe spaces becomes increasingly difficult to sustain.

At a broader level, some narratives express a profound disillusionment with modern industrial systems, framing contamination as the outcome of unsustainable patterns of production and consumption. This perspective reflects a shift from localized environmental concern to a more systemic critique, in which PFAS contamination is interpreted as part of a wider ecological crisis.

Within the framework of TET, these dynamics indicate an expansion of lifescape disruption beyond the immediate context of exposure. The environment is no longer a reliable reference point for meaning-making but becomes a source of ongoing uncertainty and moral questioning. In this sense, PFAS contamination not only alters ecological conditions but also destabilizes the symbolic and cultural frameworks through which individuals and communities relate to the environment.

#### 5.1.5. Inversion of Livelihood

This subtheme captures the disruption of everyday economic activities as a consequence of environmental contamination, reflecting how practices related to subsistence, income, and social life are reconfigured under conditions of uncertainty. Across several narratives (*n* = 12), participants describe how activities traditionally associated with sustenance, income, and social life, such as food production, fishing, and eating in local establishments, are redefined as potential pathways of exposure.

A central element emerging from the narratives is the contamination of food systems. Practices such as home gardening, small-scale farming, and animal husbandry, previously associated with health, self-sufficiency, and environmental connection, are reinterpreted as risky. As one participant explains:

*“The most contaminated people were the ones who were growing food… vegetables and fruits… and also having animals.”*

This inversion transforms subsistence activities into sources of vulnerability, undermining both material self-reliance and symbolic connections to the land. Similarly, participants report significant disruptions to local consumption practices and social life. Concerns about contaminated water and food have led some residents to avoid restaurants and other shared spaces, reflecting a broader erosion of trust in local systems of provision: “We have to quit going to all the restaurants in town… everything is affected”.

Environmental contamination also directly impacts resource-based activities such as fishing, with the imposition of consumption advisories rendering local resources unusable and affecting related economic sectors, including tourism. In these cases, environmental degradation translates into tangible economic consequences, limiting both subsistence and income-generating opportunities.

A further layer of complexity emerges in communities where local economies are historically tied to the industries responsible for contamination. Participants describe ambivalent relationships with polluting companies, which are simultaneously sources of economic stability and environmental harm. As one narrative highlights, industrial actors that contributed to local prosperity are later reinterpreted as responsible for long-term contamination, generating tensions between economic dependence and environmental accountability.

Within the framework of TET, these dynamics reflect a broader disruption of livelihood as a core component of lifescape. Contamination not only alters specific activities but reconfigures the conditions under which individuals and communities sustain themselves, materially and socially. In this sense, PFAS contamination produces a systemic form of livelihood insecurity, in which both subsistence practices and local economies are destabilized by the pervasive and enduring nature of environmental risk.

#### 5.1.6. Inversion of Trust

This subtheme captures a decline in trust in institutions and authorities as reported in participant accounts. Across a substantial portion of the narratives (*n* = 13), participants describe a shift from initial expectations of protection and accountability to experiences of disappointment, suspicion, and conflict. Trust in public institutions, regulatory agencies, and polluting industries is progressively replaced by perceptions of inaction, concealment, and inadequate response. In many accounts, this transformation begins with unmet expectations. Participants report initially relying on state authorities and responsible actors to provide information, ensure safety, and implement remediation measures. However, the perceived absence of timely intervention and transparency leads to a gradual breakdown of institutional credibility. As one participant notes: “We looked to the state… and to the polluter… but none of those things have really happened”.

This erosion of trust is further intensified by narratives describing delayed communication, lack of disclosure, and, in some cases, the minimization of risk. Participants frequently interpret these dynamics as forms of institutional failure, or even intentional withholding of information, which undermine their ability to make informed decisions about their health and environment: “They knew about these chemicals for years… the public never knew”. Such accounts contribute to what can be conceptualized as a form of institutional betrayal, in which actors responsible for safeguarding public well-being are perceived as having failed in their duties. This perception not only amplifies feelings of vulnerability but also reshapes how individuals interpret past exposure and assign responsibility.

Importantly, this loss of trust extends beyond governmental and industrial actors to include the healthcare system. Some participants report experiences of dismissal or lack of recognition from medical professionals further reinforcing a sense of abandonment: “When I presented them blood test results, one of them answered me: I’m just a surgeon. I don’t need to know about PFAS.”

Within the framework of TET, this dynamic reflects a profound disruption of lifescape, as trust in institutional structures, essential for interpreting risk and guiding action, is destabilized. At the same time, it contributes to lifestrain by intensifying stress, uncertainty, and feelings of isolation.

In this sense, PFAS contamination produces not only environmental and health impacts but also a crisis of institutional legitimacy, in which the erosion of trust becomes a central component of the lived experience of contamination.

#### 5.1.7. Environmental Stigma

This subtheme captures how environmental contamination is associated with experiences of stigma in participants’ accounts, affecting not only places but also individuals and communities. Across several narratives (*n* = 11), participants describe processes of social devaluation, marginalization, and reduced credibility associated with living in contaminated areas.

At the individual level, stigma often manifests through the delegitimization of personal experiences. Participants report being labeled as exaggerated or irrational when raising concerns about contamination, particularly in cases involving health impacts: “People looked at me as a crazy mom…”. Similarly, others describe being dismissed as “fearmongers,” reflecting a broader tendency to question the legitimacy of affected communities’ claims.

At the community level, stigma intersects with structural inequalities. Some participants highlight how marginalized populations, particularly low-income, immigrant, or minority communities, experience delayed institutional responses and reduced access to resources: “Our community is always last… and I feel it’s because we are a significant Latino community”.

These accounts suggest that environmental contamination can reinforce existing social vulnerabilities, producing uneven distributions of recognition, support, and protection. Stigma also has material consequences. Participants describe economic impacts such as reduced property values and unequal access to mitigation measures, including water testing and filtration systems, which are often financially inaccessible: “People have lost property value… they should be compensated”.

Within the framework of TET, environmental stigma represents a further extension of lifescape disruption, in which not only the environment but also the social value of affected individuals and communities is undermined. In this sense, contamination produces a form of social invisibility, where suffering is minimized, contested, or unevenly recognized.

### 5.2. Theme 2: Lifestyle

This theme captures how environmental contamination restructures everyday life through what can be conceptualized as regimes of forced adaptation. Across the narratives (*n* = 25), participants describe the transformation of ordinary routines into practices governed by risk awareness, uncertainty, and precaution. Activities that were previously habitual and taken for granted become sites of continuous evaluation and control.

A first dimension of this transformation concerns water use, which shifts from an invisible and trusted resource to a managed and potentially hazardous substance. Participants describe the strict regulation of water consumption within the household, including the exclusive use of filtered systems and the avoidance of tap water for cooking and drinking: “The kids were only allowed to drink from the reverse osmosis”. This reconfiguration reflects a broader process of domestication of risk, where households become primary sites of environmental management.

A second dimension involves food practices. Participants report a shift away from locally sourced or home-grown products, traditionally associated with health and sustainability, toward industrially processed alternatives perceived as safer. This includes changes in purchasing habits, ingredient scrutiny, and the avoidance of specific materials or cooking methods associated with PFAS exposure: “When I go to the supermarket, I always read everything…”.

In parallel, practices such as home gardening are abandoned, indicating a withdrawal from direct interaction with the local environment. This shift signals a reversal in the symbolic value of “natural” food, which becomes a potential source of risk rather than wellbeing.

A third dimension concerns the medicalization of everyday life. Some participants describe increased reliance on medical treatments, frequent health monitoring, and the integration of illness management into daily routines: “I have to take so much medication daily…”. This reflects a transition toward what can be understood as a medically structured lifestyle, where health concerns become a central organizing principle of everyday activities.

Importantly, these adaptive changes are not always temporary. Over time, they tend to stabilize, producing long-term transformations in habits, consumption patterns, and risk perception. Within TET, this process reflects lifestyle disruption, where individuals continuously reorganize their practices in response to persistent environmental uncertainty.

In some cases, these adaptations evolve into forms of collective engagement. Participants describe how managing contamination in everyday life becomes intertwined with activism, advocacy, and community mobilization: “It’s become my life now… so many people are water warriors”. This shift illustrates how forced adaptation can extend beyond individual coping, generating new forms of social participation and collective response to environmental turbulence.

### 5.3. Theme 3: Lifestrain

This theme captures the psychosocial consequences of environmental turbulence, focusing on the emotional, cognitive, and relational burdens associated with long-term exposure to PFAS contamination. Across the narratives (*n* = 18), participants describe a condition of persistent stress shaped by uncertainty, perceived lack of control, and institutional failure. In line with TET, lifestrain reflects the cumulative impact of disrupted lifescape and lifestyle, manifesting as chronic emotional distress and adaptive strain. Five interconnected subthemes emerge: shock and fear, chronic concern, anger, parental guilt, and relational strain.

#### 5.3.1. Shock and Fear

For most participants (*n* = 23), lifestrain begins with an initial phase of shock, triggered by the discovery of contamination or by the realization of its extent and duration. This moment often involves an intense emotional and embodied reaction, as individuals confront information that fundamentally challenges their assumptions about safety and protection. As one participant describes: “I could physically feel my body… in shock and awe”. This reaction is frequently linked not only to the presence of contamination but also to the perception that risks were known and not adequately communicated. The resulting emotional response combines fear with a sense of betrayal, amplifying the initial impact.

Following this initial shock, participants often describe a rapid transition into a state of ongoing distress, in which everyday life becomes structured around managing uncertainty and perceived risk. Experiences of illness within the family further intensify this condition, producing feelings of fear, vulnerability, and anticipatory anxiety: “We were terrified… I just remember a dark cloud looming over us”.

Within the framework of TET, this phase represents the acute onset of lifestrain, where emotional responses are immediate, intense, and closely tied to the disruption of core assumptions about safety, health, and institutional protection.

#### 5.3.2. Chronic Concern

Beyond the initial shock, lifestrain evolves into a more persistent condition characterized by chronic concern. Across many narratives (*n* = 18), participants describe a continuous state of worry sustained by uncertainty, incomplete knowledge, and the long-term nature of PFAS exposure. This form of concern is not simply a reaction to lack of information; rather, it emerges from the tension between increasing awareness and the absence of effective solutions. As one participant notes: “We have more information… but there are still a lot of unknowns”.

The recognition of contamination as widespread, persistent, and difficult to remediate contributes to a sense of helplessness and loss of control. Participants frequently express the perception that the situation is irreversible: “We can never get it out of the water”. This awareness fosters a form of anticipatory anxiety oriented toward uncertain future outcomes, including potential health effects that may emerge over time. Questions such as “What is going to happen next?” reflect a temporal extension of lifestrain, in which concern is projected into the future. In some cases, this ongoing uncertainty gives rise to fatalistic attitudes, where individuals perceive limited possibilities for action: “There’s nothing you can do about it”.

Within TET, this condition can be understood as chronic lifestrain, where stress becomes normalized and embedded in everyday life. Importantly, concern is not limited to individual risk but extends to future generations, amplifying its emotional and moral dimensions: “What is this going to do for future generations?”. In this sense, PFAS contamination produces a temporally extended form of distress, where uncertainty about long-term and intergenerational effects becomes a defining feature of lived experience.

#### 5.3.3. Anger

Anger emerges as a central and structured emotional response to environmental turbulence, particularly in contexts characterized by prolonged exposure and perceived institutional failure. Across multiple narratives (*n* = 15), anger is not merely reactive but takes the form of moral outrage, rooted in experiences of betrayal, neglect, and preventable harm. Participants frequently link their anger to the realization that contamination was known, minimized, or insufficiently addressed by responsible actors: “Angry that these were toxic chemicals… touted as some miracle”. This framing reflects a shift from fear-based reactions to accountability-oriented interpretations, where emotional responses are directed toward identifiable institutions.

Importantly, anger also functions as a mechanism of agency reconstruction. In situations where individuals perceive a loss of control, anger becomes a driver for information-seeking, confrontation, and engagement with public authorities: “I’m angry… I want to know”.

In several cases, this emotional response evolves into collective mobilization. Participants describe how shared outrage contributes to the formation of advocacy groups and public pressure initiatives: “Angry moms… ‘Why aren’t you doing anything?’”.

Within Environmental Turbulence Theory, anger can thus be understood as a transitional form of lifestrain, mediating the shift from passive suffering to active response. While it originates in distress, it simultaneously enables forms of resistance and social action, transforming emotional disruption into collective agency.

#### 5.3.4. Parental Guilt

A distinct dimension of lifestrain is parental guilt, which emerges as a particularly intense and morally charged response among participants with children (*n* = 17) especially mothers. This form of distress is characterized by the internalization of responsibility for harm that is, in reality, externally produced and structurally mediated. Participants describe a profound sense of having unintentionally exposed their children to danger: “I sent my children to a daycare center that had highly contaminated water”, “I had no idea that I was… poisoning my unborn son”. These narratives reflect more than emotional distress; they point to what can be conceptualized as moral injury, where deeply held identity roles, such as caregiving and protection, are perceived as compromised.

The temporal dimension is particularly salient. Guilt is often retrospective, emerging after the discovery of contamination, but it is also prospective, as parents fear long-term consequences for their children’s health and development. This dual temporality intensifies the burden of lifestrain, anchoring it both in past actions and future uncertainties.

Within TET, parental guilt represents an internalized form of lifestrain, where structural failures are translated into personal responsibility. This mechanism amplifies emotional distress by relocating the source of harm from external systems to the self, even when individuals had no access to information or capacity to prevent exposure.

#### 5.3.5. Relational Strain

Relational strain captures the erosion of social bonds and institutional relationships under conditions of environmental turbulence (*n* = 18). Participants describe a progressive weakening of trust and cohesion across multiple levels, including relationships with public institutions, local communities, and social networks.

A key dimension of this process is civic betrayal, defined as the perceived failure of authorities to fulfill their protective role. Participants express frustration and moral condemnation toward institutions that have delayed action or ignored community concerns: “My community… still doesn’t have a clean-up plan”, “The city is just ignoring it… that’s a shame”. These evaluations extend beyond dissatisfaction, reflecting a broader rupture in the social contract between citizens and governing bodies.

Relational strain also manifests horizontally, within communities themselves. Some participants report experiences of social isolation, conflict, or marginalization when raising concerns about contamination: “I’ve been blocked… for telling the truth”. In these cases, environmental risk becomes socially contested, fragmenting communities rather than uniting them. Additionally, attempts at institutional engagement are often perceived as ineffective or symbolic. Participatory mechanisms, such as advisory groups, are described as bureaucratic and unresponsive, reinforcing feelings of exclusion and disempowerment: “It turned into… just kind of a bureaucracy… it was going nowhere”.

Within TET, relational strain represents the social dimension of lifestrain, where disruption extends beyond individual experience to affect the networks and institutions that structure collective life. This process not only intensifies emotional distress but also undermines the capacity for coordinated response, further entrenching the effects of environmental turbulence.

## 6. Discussion

The findings of this study show that slow invisible chronic technological disasters such as PFAS contamination cannot be adequately understood through biomedical or environmental indicators alone. By applying the Theory of Environmental Turbulence (TET) as developed by Edelstein [39,66], the analysis demonstrates how contamination operates as a multidimensional disruption of the taken-for-granted relationships that structure everyday life, affecting lifescape, lifestyle, and lifestrain in interconnected ways. In particular, the findings show how the absence of immediate sensory markers and the delay in institutional recognition contribute to forms of uncertainty and invisibility that intensify environmental turbulence. These dynamics also resonate with broader concepts such as collective trauma [41,48,80,81] and second-order victimization [82,83], especially in contexts where affected communities experience prolonged neglect or denial.

Previous applications of TET have primarily examined contexts in which contamination was relatively bounded and identifiable in time and space, often involving more localized and temporally defined exposure scenarios [26]. The present findings extend this framework by showing that environmental turbulence can emerge not from a single catastrophic rupture but from the progressive, belated recognition of long-term exposure, thereby reflecting broader evidence that contemporary forms of pollution, such as PFAS, are diffuse, delayed, and difficult to delimit in time and space [54].

In line with TET, the results highlight how environmental contamination disrupts what Edelstein conceptualizes as cumulative normality, that is, the implicit assumptions through which individuals organize their expectations, routines, and sense of safety [66]. However, this study extends the theory by showing how, in the case of PFAS, such disruption does not emerge from a sudden catastrophic event, but from processes of delayed recognition, prolonged uncertainty, and progressive reinterpretation of everyday experience. In acute disasters, communities can identify a clear “before” and “after”, whereas in the case of PFAS, turbulence is constituted through retrospective reinterpretation: as one participant stated, “I like to say in May of 2014, my life changed forever”. The moment of recognition does not simply reveal a pre-existing hazard but fundamentally restructures the meaning of past experience. This temporal inversion represents a distinctive feature of slow disasters that TET, in its original formulation, did not fully theorize, and to which this study contributes a meaningful refinement. In this sense, environmental turbulence unfolds as a temporally extended and socially mediated process, rather than as an immediate reaction to visible environmental change.

The integration of the stage-based model of psychosocial reaction to toxic exposure [23] developed through a bottom-up approach grounded in the lived experiences of affected populations, with both TET and narrative epidemiology represents a further theoretical contribution. While TET identifies what is disrupted across lifescape, lifestyle, and lifestrain, the stage-based model illuminates how disruption unfolds over time, showing how they are articulated through participants’ narratives and revealing patterns that might remain obscured in more top-down or expert-driven approaches. The progression from initial shock, “I could physically feel my body… in shock and awe”, through chronic concern, anger, and relational strain mirrors the trajectory identified in previous research on PFAS-exposed communities in the Veneto region [23], suggesting a generalizable pattern across geographically and culturally distinct contexts. However, this potential generalizability should be interpreted with caution, as the stage-based model requires further validation across different datasets, methodological designs, and socio-institutional contexts.

Importantly, the present findings enrich this trajectory by showing that stages are not strictly linear but can overlap or recur depending on institutional responsiveness and social support. In this sense, the bottom-up model serves as a methodological bridge between the structural explanatory power of TET and the experiential richness of narrative epidemiology, enabling personal narratives to be read as collective documents that map shared patterns of exposure, reaction, and meaning-making, what Frank [61] has described as a “narrative diagnosis” of contamination.

Existing research on the psychosocial impacts of PFAS contamination has documented elevated levels of distress and anxiety in affected populations [21,22,23,24,25]. While some of them have adopted qualitative approaches to explore the lived experiences of PFAS-exposed communities [21,23], a portion of them has relied on quantitative indicators that capture the intensity of psychological distress without fully illuminating its social, symbolic, and relational dimensions [22,25]. Moreover, even qualitative contributions have tended to focus on specific populations or geographic contexts, limiting the generalizability of their findings. A broader limitation of this body of work is its tendency to frame psychosocial harm as a secondary consequence of toxicological exposure, rather than as a structurally embedded dimension of the contamination experience itself. The present study addresses this gap by showing that lifescape disruption operates across multiple interacting registers.

The findings of this study illustrate how disruptions in lifescape are manifested through processes such as the re-signification of the home as a site of exposure, the erosion of institutional trust, and the emergence of environmental stigma. The inversion of trust, for instance, is not a uniform process: the account of a participant dismissed by a physician “I’m just a surgeon. I don’t need to know about PFAS” explains how institutional betrayal extends beyond regulatory failure to encompass the healthcare system itself, a dimension largely absent from existing PFAS research [82,83]. Similarly, the transformation of home into a “site of managed risk” captured in participants’ accounts of installing filtration systems and restricting children’s water use, reflects a qualitative shift in the meaning of domestic space that epidemiological studies have largely overlooked. Also, participants’ accounts of being dismissed as “fearmongers” or ignored by authorities reflect not only individual frustration but broader processes of symbolic marginalization, in which affected communities struggle to have their experiences recognized. These dynamics reinforce the interpretation of lifescape disruption as both cognitive and relational, involving shifts in meaning, identity, and trust. Finally, the findings on environmental stigma extend existing theorizations of place-based devaluation [84] by showing that stigma operates simultaneously at individual, community, and structural levels, with unevenly distributed effects. The account of a participant who described her community as “always last… because we are a significant Latino community” illustrates how contamination reinforces pre-existing racial and socioeconomic vulnerabilities, a dimension that has received comparatively limited attention in the PFAS literature, which has often treated affected communities as relatively homogeneous [85]. Addressing this gap requires aligning environmental health research more explicitly with environmental justice frameworks [86,87]. These dynamics also resonate with the concept of second-order victimization [40,59,60], which describes how affected individuals and communities experience a compounded form of harm when their suffering is minimized, contested, or ignored by institutions and the broader public. The present findings extend this concept by showing that second-order victimization in PFAS-contaminated communities operates not only through explicit denial but also through more subtle mechanisms, such as medical dismissal, bureaucratic unresponsiveness, and the stigmatization of community advocates. In this sense, second-order victimization becomes structurally embedded in the contamination experience, amplifying lifestrain and further eroding institutional trust [54,85].

At the level of lifestyle, the study shows how contamination leads to the reorganization of everyday practices, including water use, food consumption, and domestic routines. These changes are not merely adaptive behaviors [23], but continuous reminders of risk that embed environmental turbulence into daily life. The need to constantly monitor and modify ordinary activities reflects a form of ongoing exposure that extends beyond the material presence of contaminants, shaping how individuals inhabit and interact with their environment.

Finally, lifestrain emerges as a form of persistent psychosocial strain [23,54] characterized by shock, chronic concern, anger, parental guilt, and relational tensions. Importantly, these responses should not be understood as isolated emotional reactions, but as socially structured and morally grounded responses to perceived injustice and institutional failure. For instance, expressions of anger reflect not only distress but also moral evaluations of responsibility, while parental guilt illustrates the internalization of harm in contexts where exposure was neither visible nor avoidable. In this sense, lifestrain captures the cumulative psychological burden of living within conditions of uncertainty and limited control.

This study demonstrates that narrative testimonies constitute an independent epistemic resource revealing patterns of harm invisible to conventional methods [88]. For instance, parental guilt, described by participants as a form of moral injury: “I had no idea that I was… poisoning my unborn son”, involves a specific configuration of identity, temporality, and responsibility that standardized distress measures cannot capture. By framing it as an internalized form of lifestrain in which structural failures are displaced onto individual caregivers, this study contributes a more nuanced understanding of how environmental contamination produces harm that is simultaneously personal and political. Furthermore, the analysis of anger as a transitional form of lifestrain, mediating the shift from passive suffering to collective mobilization, offers a theoretical refinement that existing research has not fully developed. When grounded in moral reasoning [89] and directed toward identifiable institutional actors, anger can function as a catalyst for advocacy and collective action, suggesting that emotional responses to contamination should be understood not only as indicators of harm but as potential resources for community resilience.

Within this framework, narrative processes emerge as a central mechanism through which individuals and communities interpret, communicate, and respond to environmental disruption. In this sense, the archive Living With PFAS itself functions as a site of collective memory [40,59,60], preserving testimonies that might otherwise remain invisible in official narratives. The act of sharing and archiving personal accounts contributes to the construction of a shared historical record that counters institutional silence and sustains public awareness of long-term environmental harm across generations. This dimension is particularly significant in the case of PFAS, where the absence of a clearly identifiable catastrophic event limits the incorporation of contamination experiences into broader cultural and public memory.

In line with the perspective of narrative epidemiology and cultural memory studies narratives contribute to the construction of shared meanings, the attribution of responsibility, and the development of collective responses [90,91,92]. At the same time, the invisibility and delayed recognition of PFAS contamination limit the incorporation of these experiences into broader public memory, contributing to their marginalization. This highlights the importance of narrative work in making visible forms of harm that remain socially and institutionally unrecognized.

The integration of TET [39] with Psycho-Social Impact Assessment (PSIA) [65] further strengthens the analytical contribution of this study. In particular, PSIA enables the translation of narrative evidence into analytically structured dimensions that can inform public health assessment and intervention, making it possible to systematically identify otherwise hidden forms of psychosocial harm. While TET provides the theoretical framework for understanding environmental disruption, PSIA offers an applied approach for identifying and assessing its psychosocial consequences across different phases of contamination. Together, they create a bridge between theoretical interpretation and empirical assessment, enabling a more comprehensive understanding of the impacts of slow and invisible environmental disasters.

Overall, the findings highlight the need for approaches that integrate epidemiological, psychosocial, and narrative dimensions in order to fully capture the complexity of environmental harm. In the case of PFAS, environmental turbulence emerges not only from toxic exposure but from processes of uncertainty, delayed recognition, and institutional response, all of which shape how individuals experience and make sense of contamination over time.

## 7. Limitations

This study presents several limitations that should be acknowledged in interpreting the findings.

First, the analysis is based on a relatively limited number of narratives, which, while sufficient for qualitative inquiry, does not aim at statistical representativeness [93]. The objective of the study is instead to explore meaning-making processes and psychosocial dynamics associated with PFAS contamination.

Second, the dataset consists of secondary narrative data drawn from a voluntary archive of testimonies. As such, the researchers did not have direct control over data generation, including interview structure, probing depth, or contextual clarification. This may limit the consistency and comparability of narratives, as well as the possibility of exploring emergent themes in greater depth [94,95].

Third, the voluntary nature of participation introduces a self-selection bias, as individuals who are more affected, engaged, or motivated may be overrepresented [96,97]. Consequently, the findings may reflect more intense or articulated experiences of contamination, while less visible or less vocal perspectives remain underrepresented.

Fourth, the group of participants is heterogeneous and spans different geographical and socio-cultural contexts, which, while enriching the diversity of perspectives, may also limit the context-specific interpretability of certain findings [98]. It is also worth noting that 24 out of 25 narratives originate from North American contexts, with only one testimony from Italy. While this reflects the composition of the Living With PFAS archive, it may limit the transferability of the findings to other regulatory, cultural, and institutional settings, particularly in European or Global South contexts where PFAS contamination is also documented but institutional responses and community dynamics may differ substantially.

Fifth, although the findings highlight the intersection of environmental contamination with racial and socioeconomic inequalities, the study was not designed as an environmental justice inquiry and does not systematically address the structural determinants of unequal exposure and recognition [99]. Future research should explicitly integrate environmental justice frameworks to examine how pre-existing social vulnerabilities shape the experience of PFAS contamination across different communities and institutional contexts.

Sixth, the cross-sectional nature of the data does not allow for a full examination of the temporal evolution of psychosocial responses [100]. Although narratives often contain retrospective elements, they do not substitute for longitudinal observation of how lifescape, lifestyle, and lifestrain transform over time.

Despite these limitations, the narrative approach adopted in this study provides rich and in-depth insights into the lived experience of environmental contamination, allowing for the identification of patterned psychosocial processes that are often overlooked in quantitatively oriented research [66].

Future research could address these limitations by adopting mixed-method and longitudinal designs, combining narrative analysis with primary data collection to enhance depth and comparability. Expanding the diversity of participants, including less visible or less engaged populations, would further strengthen the understanding of how PFAS contamination is experienced across different social groups. Additionally, comparative studies across national and regulatory contexts could help clarify the role of institutional and cultural factors in shaping environmental turbulence. Finally, longitudinal research would be particularly valuable in examining the evolution of collective memory, risk perception, and psychosocial adaptation over time, contributing to a more dynamic understanding of slow environmental disasters. In particular, while the stage-based model proposed in this study offers a useful interpretative framework, its applicability across different contexts should be further tested through comparative and longitudinal research designs.

## 8. Conclusions

This study demonstrates that slow invisible chronic technological disasters, such as PFAS contamination, cannot be adequately understood through biomedical or environmental metrics alone. By applying the Theory of Environmental Turbulence (TET) to narrative data, the findings show how contamination operates as a multidimensional disruption that reconfigures lifescape, lifestyle, and lifestrain. In this sense, environmental harm emerges not only as material exposure, but as a process that destabilizes everyday routines, social relationships, and systems of meaning.

By grounding the analysis in a bottom-up, narrative-based approach, this study not only applies but empirically refines TET, showing how its core dimensions emerge from the lived experiences and meaning-making processes of affected individuals rather than being imposed a priori. In doing so, the study demonstrates the analytical value of bottom-up models in capturing dimensions of environmental turbulence that remain underexplored in more top-down or expert-driven approaches.

The results highlight that psychosocial responses to PFAS contamination are not isolated reactions, but patterned processes—including inversion of trust, environmental stigma, chronic concern, and moral emotions such as anger and parental guilt—that evolve across interconnected domains of life.

Within this framework, the integration of TET with Psycho-Social Impact Assessment (PSIA) represents a key contribution of this study. While TET provides the theoretical architecture for understanding environmental disruption, PSIA operationalizes these insights into an applied framework capable of identifying and assessing the psychosocial impacts of contamination across different temporal phases. This combined approach offers a bridge between theory and practice, making visible forms of harm that are often overlooked in conventional risk assessment and policy responses.

More broadly, the findings underscore the importance of incorporating narrative and experiential knowledge into the study of environmental crises. Narratives do not simply describe harm; they actively contribute to the construction of collective awareness, the articulation of responsibility, and the emergence of community agency. In contexts characterized by delayed recognition and institutional silence, narrative processes become central to both understanding and responding to environmental injustice.

These insights point to the need for a transdisciplinary and longitudinal approach to chronic contamination, one that integrates epidemiological, psychosocial, and narrative dimensions. Such an approach is essential not only for capturing the full scope of environmental harm, but also for designing more responsive, inclusive, and just policy interventions.

Ultimately, recognizing and addressing the psychosocial dimensions of environmental turbulence is a necessary step toward restoring trust, rebuilding collective life, and advancing environmental justice in communities affected by long-term contamination.

## Figures and Tables

**Table 1:** Narratives of PFAS exposure with source of contamination.

Narrative	Gender of Participant	Contamination Exposure
N.1	M	Rockford, Michigan: He worked for 31 years at Wolverine Worldwide, a footwear company that used PFAS in its manufacturing process
N.2	F	Portsmouth, New Hampshire: In 2014 she discovered that the drinking water in her community was contaminated with elevated levels of PFAS
N.3	F	Wilmington, North Carolina: In 2017 she discovered that she had been exposed to PFAS through contaminated water
N.4	F	Oscoda, Michigan: Exposed through a former military air base where PFAS compounds were widely used in firefighting foams
N.5	F	Leland, North Carolina: Exposed after an upstream chemical plant released large amounts of PFAS into the Cape Fear River, source of drinking water for her area
N.6	M	Grayling, Michigan: Resident of a town heavily contaminated with PFAS, likely due to military activities in the area
N.7	M	Rockford, Michigan: He and his family drank PFAS-contaminated municipal water for about a decade
N.8	M	U.S. Air Force firefighter who was extensively exposed to PFAS-containing firefighting foams during his service between 1991 and 2017
N.9	M	Plainfield Township, Michigan: Resident of a community subjected to PFAS contamination caused by the Wolverine company
N.10	M	Belding, Michigan
N.11	F	Veneto, Italia
N.12	F	Hoosick Falls, New York
N.13	F	Davis-Monthan Air Force Base, Tucson, Arizona
N.14	F	Rockford, Michigan
N.15	F	Wilmington, North Carolina
N.16	F	Grand Rapids, Michigan
N.17	F	Belmont, Michigan
N.18	F	Plainfield Township, Michigan
N.19	F	Kalamazoo, Michigan
N.20	M	Kalamazoo, Michigan
N.21	F	Rockford, Michigan
N.22	F	Belmont, Michigan
N.23	M	Grand Rapids, Michigan
N.24	M	Oscoda, Michigan
N.25	F	Merrimack, New Hampshire: Resident of a community where a company called Saint-Gobain released PFAS chemicals into the environment for decades, contaminating both the water and the soil

**Table 2:** Themes and sub-themes emerged.

Theme	Sub-Theme
lifescape	inversion of health
	inversion of self
	inversion of home, community and place
	inversion of environment
	inversion of livelihood
	inversion of trust
	environmental stigma
lifestyle	
lifestrain	shock and fear
	chronic concern
	anger
	parental guilt
	relation strain

## Data Availability

Data is available from the authors upon reasonable request.

## References

[1] Eskenazi B., Warner M., Brambilla P., Signorini S., Ames J., Mocarelli P. (2018). The Seveso Accident: A Look at 40 years of Health Research and Beyond. Environ. Int..

[2] Consonni D., Rognoni M., Cavalieri d’Oro L., Pesatori A.C. (2024). Mortality and Cancer Incidence in a Population Exposed to TCDD after the Seveso, Italy, Accident (1976–2013). Occup. Environ. Med..

[3] Scheringer M., Arp H.P.H., Cousins I.T. (2026). Boundaries, Limits, Global Threats—How Can the Impacts of Global Synthetic Pollutants Be Reduced?. Environ. Sci. Technol..

[4] Bernhardt E.S., Rosi E.J., Gessner M.O. (2017). Synthetic Chemicals as Agents of Global Change. Front. Ecol. Environ..

[5] OECD (2022). Fact Cards of Major Groups of Per- and Polyfluoroalkyl Substances (PFASs).

[6] OECD (2018). Summary Report on the New Comprehensive Global Database of Per- and Polyfluoroalkyl Substances (PFASs).

[7] Zindel H., Powers M., Brown P., Cordner A. (2021). State Messaging on Toxic Chemical Exposure: Per- and Polyfluoroalkyl Substances and the Individualization of Risk on State Websites in the United States. Environ. Commun..

[8] Liu Z., Yang J.Z. (2023). Communicating Per- and Polyfluoroalkyl Substances (PFAS) Contamination to the Public Through Personal Relevance. J. Health Commun..

[9] Bonato T., Pal T., Benna C., Di Maria F. (2025). Contamination of the Terrestrial Food Chain by Per- and Polyfluoroalkyl Substances (PFAS) and Related Human Health Risks: A Systematic Review. Sci. Total Environ..

[10] Zhou Y., Wang X., Wang C., Ji Z., Niu X., Dong H. (2024). Fate of “forever Chemicals” in the Global Cryosphere. Earth Sci. Rev..

[11] Frisbee S.J., Brooks A.P., Maher A., Flensborg P., Arnold S., Fletcher T., Steenland K., Shankar A., Knox S.S., Pollard C. (2009). The C8 Health Project: Design, Methods, and Participants. Environ. Health Perspect..

[12] Sunderland E.M., Hu X.C., Dassuncao C., Tokranov A.K., Wagner C.C., Allen J.G. (2019). A Review of the Pathways of Human Exposure to Poly- and Perfluoroalkyl Substances (PFASs) and Present Understanding of Health Effects. J. Expo. Sci. Environ. Epidemiol..

[13] Cindy S.L.Y., Alrazihi L.A., Wong S.T., Wong S.F. (2025). Per- and Poly-Fluoroalkyl Substances (PFAS) and Human Health: A Review of Exposure Routes and Potential Toxicities across the Lifespan. Environ. Toxicol. Chem..

[14] Liu B., Zhu L., Wang M., Sun Q. (2023). Associations between Per- and Polyfluoroalkyl Substances Exposures and Blood Lipid Levels among Adults-A Meta-Analysis. Environ. Health Perspect..

[15] Boston C., Keck S., Naperala A., Collins J. (2025). The Evolution of PFAS Epidemiology: New Scientific Developments Call into Question Alleged “Probable Links” between PFOA and Kidney Cancer and Thyroid Disease. Front. Public Health.

[16] Connolly J.C., Ishihara Y., Sawaya E., Whitfield V., Garrity N., Sohata R., Tsymbal M., Lundberg A., La Merrill M.A., DeWitt J.C. (2025). Per- and Polyfluoroalkyl Substances (PFAS) Enhance Cholesterol Accumulation and Dysregulate Inflammatory Responses in Macrophages. Cardiovasc. Toxicol..

[17] Seyyedsalehi M.S., Boffetta P. (2023). Per- and Poly-Fluoroalkyl Substances (PFAS) Exposure and Risk of Kidney, Liver, and Testicular Cancers: A Systematic Review and Meta-Analysis. Med. Lav..

[18] Gaillard L., Barouki R., Blanc E., Coumoul X., Andréau K. (2025). Per- and Polyfluoroalkyl Substances as Persistent Pollutants with Metabolic and Endocrine-Disrupting Impacts. Trends Endocrinol. Metab..

[19] Qu R., Wang J., Li X., Zhang Y., Yin T., Yang P. (2024). Per- and Polyfluoroalkyl Substances (PFAS) Affect Female Reproductive Health: Epidemiological Evidence and Underlying Mechanisms. Toxics.

[20] Li L., Guo Y., Ma S., Wen H., Li Y., Qiao J. (2024). Association between Exposure to Per- and Perfluoroalkyl Substances (PFAS) and Reproductive Hormones in Human: A Systematic Review and Meta-Analysis. Environ. Res..

[21] Calloway E.E., Chiappone A.L., Schmitt H.J., Sullivan D., Gerhardstein B., Tucker P.G., Rayman J., Yaroch A.L. (2020). Exploring Community Psychosocial Stress Related to Per- and Poly-Fluoroalkyl Substances (PFAS) Contamination: Lessons Learned from a Qualitative Study. Int. J. Environ. Res. Public Health.

[22] Lazarevic N., Smurthwaite K.S., Batterham P.J., Lane J., Trevenar S.M., D’Este C., Clements A.C.A., Joshy A.L., Hosking R., Gad I. (2023). Psychological Distress in Three Australian Communities Living with Environmental Per- and Polyfluoroalkyl Substances Contamination. Sci. Total Environ..

[23] Menegatto M., Lezzi S., Musolino M., Zamperini A. (2022). The Psychological Impact of Per- and Poly-Fluoroalkyl Substances (PFAS) Pollution in the Veneto Region, Italy: A Qualitative Study with Parents. Int. J. Environ. Res. Public Health.

[24] Schmitt H.J., Calloway E.E., Sullivan D., Clausen W., Tucker P.G., Rayman J., Gerhardstein B. (2021). Chronic Environmental Contamination: A Systematic Review of Psychological Health Consequences. Sci. Total Environ..

[25] Scharnetzki E., Rokoff L.B., Senechal K., Bosquet Enlow M., Fleisch A.F., Criswell R. (2026). Psychosocial Distress among Individuals Residing in a Rural PFAS-Contaminated Community. Int. J. Hyg. Environ. Health.

[26] Edelstein M.R. (1988). Contaminated Communities: The Social and Psychological Impacts of Residential Toxic Exposure.

[27] Gao C.X., Menssink J., Campbell T.C.H., Smith C.L., Ikin J.F., Lane T., Abramson M.J., Carroll M. (2023). Somatic Symptoms, Psychological Distress and Trauma after Disasters: Lessons from the 2014 Hazelwood Mine Fire and 2019–2020 Black Summer Bushfires. BMC Public Health.

[28] Zamperini A., Menegatto M. (2021). Cattive Acque.

[29] Fowlkes M., Miller P. (1987). Chemicals and Community at Love Canal.

[30] Gill D.A., Mix T.L. (2020). Love Canal: A Classic Case Study of a Contaminated Community. An Introduction to Interdisciplinary Toxicology.

[31] Bilott R. (2020). Exposure: Poisoned Water, Corporate Greed, and One Lawyer’s Twenty-Year Battle Against Dupont.

[32] Hall S.M., Zhang S., Tait G.H., Hoffman K., Collier D.N., Hoppin J.A., Stapleton H.M. (2023). PFAS Levels in Paired Drinking Water and Serum Samples Collected from an Exposed Community in Central North Carolina. Sci. Total Environ..

[33] Garrett K.K., Mok K., Brown P., Schaider L., Powers M., Fitzstevens M., Amico A., Osimo C., Cordner A., Carignan C. (2025). REACHing for PFAS Solutions: How Two Communities Responded to Drinking Water Contamination. J. Environ. Stud. Sci..

[34] Menegatto M., Zamperini A. (2023). Health and Psychological Concerns of Communities Affected by Per- and Poly-Fluoroalkyl Substances: The Case of Residents Living in the Orange Area of the Veneto Region. Int. J. Environ. Res. Public Health.

[35] Menegatto M., Zamperini A. (2024). Contamination of Perfluoroalkyl Substances and Environmental Fight for Safe and Health: The MammeNoPfas Movement as Epistemic Community. Soc. Sci..

[36] Cordner A., Brown P., Cousins I.T., Scheringer M., Martinon L., Dagorn G., Aubert R., Hosea L., Salvidge R., Felke C. (2024). PFAS Contamination in Europe: Generating Knowledge and Mapping Known and Likely Contamination with “Expert-Reviewed” Journalism. Environ. Sci. Technol..

[37] Gebbink W.A., van Leeuwen S.P.J. (2020). Environmental Contamination and Human Exposure to PFASs near a Fluorochemical Production Plant: Review of Historic and Current PFOA and GenX Contamination in the Netherlands. Environ. Int..

[38] Radke E.G., Wright J.M., Christensen K., Lin C.J., Goldstone A.E., Lemeris C., Thayer K.A. (2022). Epidemiology Evidence for Health Effects of 150 Per- and Polyfluoroalkyl Substances: A Systematic Evidence Map. Environ. Health Perspect..

[39] Edelstein M. (2018). Contaminated Communities: Coping with Residential Toxic Exposure.

[40] Halbwachs M. (1950). La Mémoire Collective.

[41] Hirschberger G. (2018). Collective Trauma and the Social Construction of Meaning. Front. Psychol..

[42] Zamperini A. (2023). Violenza Invisibile. Giulio Einaudi Editore—Piccola Biblioteca Einaudi Ns. Einaudi. https://www.einaudi.it/catalogo-libri/psicologia/psicologia-psicologia/violenza-invisibile-adriano-zamperini-9788806258634/.

[43] Ojala V. (2026). Chornobyl Visual Lexicon: Exploring the Visual Framing of Toxic Heritage from the Point of View of Participatory Culture. Vis. Stud..

[44] Thankappan A., Ponnambath H.S.K. (2026). Tracing Forty Years of Research on the Bhopal Gas Tragedy: Trends, Themes, and Future Directions. Int. J. Disaster Risk Manag..

[45] Aldeli N., Murphy D., Hanano A. (2024). Impact of Dioxins on Reproductive Health in Female Mammals. Front. Toxicol..

[46] Bertazzi P.A. (1991). Long-Term Effects of Chemical Disasters. Lessons and Results from Seveso. Sci. Total Environ..

[47] Eskenazi B., Ames J., Rauch S., Signorini S., Brambilla P., Mocarelli P., Siracusa C., Holland N., Warner M. (2021). Dioxin Exposure Associated with Fecundability and Infertility in Mothers and Daughters of Seveso, Italy. Hum. Reprod..

[48] Alexander J.C. (2012). Trauma: A Social Theory.

[49] Levine A. (1982). Love Canal: Science, Politics, and People.

[50] Newman R. (2024). Love Canal: A Toxic History. Encyclopedia of Technological Hazards and Disasters in the Social Sciences.

[51] Valsecchi S., Rusconi M., Mazzoni M., Viviano G., Pagnotta R., Zaghi C., Serrini G., Polesello S. (2015). Occurrence and Sources of Perfluoroalkyl Acids in Italian River Basins. Chemosphere.

[52] Ingelido A.M., Abballe A., Gemma S., Dellatte E., Iacovella N., De Angelis G., Zampaglioni F., Marra V., Miniero R., Valentini S. (2018). Biomonitoring of Perfluorinated Compounds in Adults Exposed to Contaminated Drinking Water in the Veneto Region, Italy. Environ. Int..

[53] Pitter G., Da Re F., Canova C., Barbieri G., Zare Jeddi M., Daprà F., Manea F., Zolin R., Bettega A.M., Stopazzolo G. (2020). Serum Levels of Perfluoroalkyl Substances (PFAS) in Adolescents and Young Adults Exposed to Contaminated Drinking Water in the Veneto Region, Italy: A Cross-Sectional Study Based on a Health Surveillance Program. Environ. Health Perspect..

[54] Onencan A.M., Bisschop L., Hendlin Y. (2024). Experiences of Living within PFAS-Polluted Environs: A Systematic Review. Crime Law Soc. Change.

[55] Erikson K. (1994). A New Species of Trouble: Explorations in Disaster, Trauma, and Community.

[56] Pemberton A., Mulder E. (2025). Bringing Injustice Back in: Secondary Victimization as Epistemic Injustice. Criminol. Crim. Justice.

[57] Bruner J. (1991). The Narrative Construction of Reality. Crit. Inq..

[58] Timmerman P., Etkin D. Narratives of Disaster: Sensemaking in Crisis. https://www.preventionweb.net/publication/narratives-disaster-sensemaking-crisis.

[59] Assmann J. (2011). Cultural Memory and Early Civilization: Writing, Remembrance, and Political Imagination.

[60] Lopez C., van Alphen F., Carmona L. (2025). Narratives and Collective Memory. Curr. Opin. Psychol..

[61] Frank A.W. (2013). The Wounded Storyteller: Body, Illness, and Ethics.

[62] Charon R. (2006). Narrative Medicine: Honoring the Stories of Illness.

[63] Baker E., Barlow C.F., Daniel L., Morey C., Bentley R., Taylor M.P. (2024). Mental Health Impacts of Environmental Exposures: A Scoping Review of Evaluative Instruments. Sci. Total Environ..

[64] Roberts M., Colley K., Currie M., Eastwood A., Li K.-H., Avery L.M., Beevers L.C., Braithwaite I., Dallimer M., Davies Z.G. (2023). The Contribution of Environmental Science to Mental Health Research: A Scoping Review. Int. J. Environ. Res. Public Health.

[65] Edelstein M.R., Sergio M., Helen R., Will R., Cave B., Viliani F., Winkler M.S. (2026). Psychosocial Impacts in Health Impact Assessment. Handbook of Health Impact Assessment.

[66] Edelstein M.R., Vanclay F., Vanclavy F., Esteves A.M. (2024). Psychosocial Impacts. Handbook of Social Impact Assessment and Management.

[67] Vanclay F. (2002). Conceptualising Social Impacts. Environ. Impact Assess. Rev..

[68] Wolf C.P. (1982). Social Impact Assessment. Impact Assess..

[69] Caggiano H., Weber E.U. (2023). Advances in Qualitative Methods in Environmental Research. Annu. Rev. Environ. Resour..

[70] Lloyd S., Gifford R. (2024). Qualitative Research and the Future of Environmental Psychology. J. Environ. Psychol..

[71] Ngwenya S., Mashau N.S., Mudau A.G., Mhlongo S.E., Traoré A.N. (2024). Community Perceptions on Health Risks Associated With Toxic Chemical Pollutants in Kwekwe City, Zimbabwe: A Qualitative Study. Environ. Health Insights.

[72] Living with PFAS. https://www.pfasstories.org.

[73] Living with PFAS Interviews Digital Collections. https://digitalcollections.library.gvsu.edu/collections/show/53.

[74] Braun V., Clarke V., Cooper H., Coutanche M.N., McMullen L.M., Panter A.T., Rindskopf D., Sher K.J. (2023). Thematic Analysis. APA Handbook of Research Methods in Psychology: Research Designs: Quantitative, Qualitative, Neuropsychological, and Biological.

[75] Patton M.Q. (2002). Qualitative Research & Evaluation Methods.

[76] Braun V., Clarke V. (2006). Using Thematic Analysis in Psychology. Qual. Res. Psychol..

[77] Halpin M., Richard N. (2021). An Invitation to Analytic Abduction. Methods Psychol..

[78] Timmermans S., Tavory I. (2022). Data Analysis in Qualitative Research: Theorizing with Abductive Analysis.

[79] Zendy K.P. (2022). Inductive/Deductive Hybrid Thematic Analysis in Mixed Methods Research. J. Mix. Methods Res..

[80] Abrutyn S. (2024). The Roots of Social Trauma: Collective, Cultural Pain and Its Consequences. Soc. Ment. Health.

[81] Niyonsenga J., Jansen S., Rutembesa E., Hermans E., Monacelli N., Caricati L. (2025). Resilience Frameworks, Measurement Tools, and Transmission Processes in the Context of Man-Made Collective Trauma: A Meta-Synthesis and Multilevel Meta-Analysis. Eur. J. Psychotraumatol..

[82] Christl M.-E., Pham K.-C.T., Rosenthal A., DePrince A.P. (2024). When Institutions Harm Those Who Depend on Them: A Scoping Review of Institutional Betrayal. Trauma Violence Abuse.

[83] Smith C.P., Freyd J.J. (2014). Institutional Betrayal. Am. Psychol..

[84] Goffman E. (1963). Stigma: Notes on the Management of Spoiled Identity.

[85] Sukhram S.D., Kim J., Musovic S., Anidugbe A., Corte E., Ahsan T., Rofail S., Mesquita N., Padilla M. (2025). PFAS Exposure, Mental Health, and Environmental Justice in the United States: Impacts on Marginalized Communities. Int. J. Environ. Res. Public Health.

[86] Mueller R., Salvatore D., Brown P., Cordner A. (2024). Quantifying Disparities in Per- and Polyfluoroalkyl Substances (PFAS) Levels in Drinking Water from Overburdened Communities in New Jersey, 2019–2021. Environ. Health Perspect.

[87] Smalling K.L., Bradley P.M. (2024). Invited Perspective: Per- and Polyfluoroalkyl Substances in Drinking Water-Disparities in Community Exposures Based on Race and Socioeconomic Status. Environ. Health Perspect..

[88] Ratcliffe E., Ogunbode C., Wilkie S., Jones C., Devine-Wright P., Uzzell D., Canter D., Korpela K., Carvalho L., Staats H. (2023). On the Importance of Qualitative Research in Environmental Psychology. J. Environ. Psychol..

[89] Zhang J., Xiong Z., Zheng H., Ma X. (2024). The Moral Psychological Justification of Anger: An Exploration of Self-Respect and Recognition. Behav. Sci..

[90] Greenhalgh T., Misak C., Payne R., Swann N. (2024). Patient Involvement in Developing Clinical Guidelines. BMJ.

[91] Greenhalgh T. (1999). Narrative Based Medicine: Narrative Based Medicine in an Evidence Based World. BMJ.

[92] Erll A. (2026). Transnational Memory. Curr. Opin. Psychol..

[93] Braun V., Clarke V. (2023). Is Thematic Analysis Used Well in Health Psychology? A Critical Review of Published Research, with Recommendations for Quality Practice and Reporting. Health Psychol. Rev..

[94] SAGE Secondary Data Analysis. https://us.sagepub.com/en-us/nam/sage-secondary-data-analysis/book237794.

[95] Kern F.G., Mustasilta K. (2023). Beyond Replication: Secondary Qualitative Data Analysis in Political Science. Comp. Political Stud..

[96] Callegaro M., Manfreda K.L., Vehovar V. (2015). Web Survey Methodology.

[97] Spencer N.H., Syrdal D.S., Coates M.L., Huws U. (2022). Assessing Bias in Online Surveys Using Alternative Survey Modes. Work Organ. Labour Glob..

[98] Jager J., Xia Y., Putnick D.L., Bornstein M.H. (2025). Improving Generalizability of Developmental Research Through Increased Use of Homogeneous Convenience Samples: A Monte-Carlo Simulation. Dev. Psychol..

[99] Gao C., Sanchez K.M., Lovinsky-Desir S. (2023). Structural and Social Determinants of Inequitable Environmental Exposures in the United States. Clin. Chest Med..

[100] Kovacs F.M., Seco-Calvo J. (2024). Limitations of a Cross-Sectional Correlation Study. Comment on Elabd et al. Prediction of Back Disability Using Clinical, Functional, and Biomechanical Variables in Adults with Chronic Nonspecific Low Back Pain. J. Clin. Med..

